# Evaluation of Smoking Cessation Advice in a Maltese Mental Health Community Clinic

**DOI:** 10.1192/j.eurpsy.2022.714

**Published:** 2022-09-01

**Authors:** N. Cortis, A. Pace, G. Grech

**Affiliations:** 1 Mount Carmel Hospital, Psychiatry, Attard, Malta; 2 Mount Carmel Hospital, Mount Carmel Hospital, Attard, Malta

**Keywords:** Cessation, Advice, smoking

## Abstract

**Introduction:**

Tobacco smoking is one of the leading causes of preventable morbidity and mortality worldwide (WHO, 2020). Smoking cessation campaigns have been effective at reducing smoking in the general population, but not in individuals with mental illness (Lê Cook et al., 2014). A downward trend in smoking has been noted in EU countries but smoking rates have remained stable in Malta (Country Health Profile, 2019).

**Objectives:**

This audit aims to assess smoking status, provision of smoking cessation advice and psychotropic dose adjustment depending on smoking status by the Bormla Mental Health Team.

**Methods:**

Patient health records were reviewed for patient demographics, psychiatric diagnosis, medical co-morbidities, smoking status and cessation advice and changes in psychiatric medication according to smoking status.

**Results:**

Of the 171 patients studied, 35% (n=61) were smokers, 33% (n=58) were non-smokers while in 30% (n=52) the smoking status was undocumented. Smokers had a mean age of 50 years with an almost equal gender distribution (49% (n=30) male and 51% (n=31) female). The most common documented psychiatric diagnoses were depression (52.5% (n=32)) and anxiety (34.5% (n=21)), while 59% (n=36) had documented medical co-morbidities. Only 14% (n=9) where given smoking cessation advice and one patient was referred to the smoking cessation clinic. One third of smokers (n=20) were prescribed psychotropic medications which are affected by smoking status but only two patients had their doses adjusted.

**Conclusions:**

Improved smoking cessation advice, referral to services, consideration of smoking cessation while prescribing and documentation are need to better patient care.

**Disclosure:**

No significant relationships.

